# Study protocol of the OrkA project: orofacial and communicative activation in old age– a cluster randomized prevention study in long-term care facilities in Lower Saxony, Germany

**DOI:** 10.1186/s12877-024-04809-5

**Published:** 2024-02-22

**Authors:** Wenke Walther, Martin Ptok, Klaus Hager, Simone Miller

**Affiliations:** 1 Institute of General Practice and Palliative Care, Hannover Medical School, Carl-Neuberg-Straße 1, 30625 Hannover, Germany; 2 Department of Phoniatrics and Pediatric Audiology of the Department of Otolaryngology, Hannover Medical School, Carl-Neuberg-Straße 1, 30625 Hannover, Germany; 3 Emeritus of the Department of Phoniatrics and Pediatric Audiology, Hannover Medical School, Carl-Neuberg-Str. 1, Hannover, Germany

**Keywords:** Prevention, Group training, Swallowing, Communication, Cluster-randomized trial, Cognition, Long-term care

## Abstract

**Background:**

The process of aging involves numerous changes in the body, influencing physical, mental, and emotional well-being. Age-related changes and degradation can impact various functions of the swallowing process and lead to delayed word retrieval. Individuals with limited linguistic stimulation may experience a more rapid decline in cognitive performance. Thus, this project explores a preventive training program targeting swallowing and linguistic-communicative skills, aimed at preserving the social participation of older individuals residing in nursing homes.

**Methods:**

A preventive intervention program, combining orofaciopharyngeal and linguistic-communicative components, will be offered twice weekly over 12 weeks in long-term care facilities in the greater Hanover area. The program will aim at: (a) activating sensitive and motor skills in the orofaciopharyngeal area to counter age-related swallowing disorders, and (b) enhancing communicative abilities through semantic-lexical activation. A cluster randomized controlled trial will be conducted to investigate whether the intervention program improves swallowing skills in older adults. Additionally, a secondary analysis will explore the impact on language skills and social participation, as well as program acceptance.

**Discussion:**

The results will provide valuable insight into the effectiveness of preventive measures addressing swallowing and speech issues in older individuals.

**Trial registration:**

The trial was registered with DRKS (German register for clinical trials) in June 2023 (study ID: DRKS00031594) and the WHO International Clinical Trail Registry Platform (secondary register).

**Supplementary Information:**

The online version contains supplementary material available at 10.1186/s12877-024-04809-5.

## Background

Aging is a complex phenomenon influenced by various factors, including genetics, lifestyle, the environment, and health conditions. As individuals age, their bodies undergo numerous changes that affect their physical, mental, and emotional well-being. Understanding the variables associated with the aging process is crucial for the development of strategies to promote “healthy aging” and to prevent age-related diseases.

The ability to swallow sufficiently and safely is not only a primary human need, but also an important aspect of quality of life and participation. Impaired swallowing can lead to malnutrition, as well as aspiration pneumonia. Pneumonia, in turn, ranks among the ten most frequent causes of death in Germany, across all age groups [1]. The act of swallowing comprises oral, pharyngeal, and esophageal phases [2], and age-related changes and degradation impact various functions within these phases. Senescence is often associated with reduced muscle strength, affecting the muscles of the mouth, pharynx, and larynx, as well as the necessary protective reflexes [3]. Dysphagia is diagnosed in 50–60% of all long-term care (LTC) facility residents, and aspiration pneumonia is the fourth most common cause of death in individuals aged 65 and older [4]. These figures underscore the significant need for preventive measures to benefit both aging individuals and the health care system more generally, in terms of cost savings.

Communication is a basic social function that allows individuals to express their needs. Added to this, social interaction is crucial for maintaining psychological well-being. Social interaction and communication both play a role in maintaining cognitive abilities. While it is commonly believed that daily language use results in well-trained linguistic abilities throughout life, it has been observed that, with advanced age, words that are used less frequently are not as easily remembered and require more cues and time for retrieval [4, 5]. Furthermore, studies have shown that cognitive performance declines more rapidly in individuals with limited linguistic stimulation [6]. Declining linguistic abilities are also early indicators of cognitive decline processes, such as those characterizing Alzheimer’s dementia [6].

In recent years, responsibility for the care and support of older individuals has increasingly shifted to LTC facilities [7–9]. Studies have identified correlations between a physically and cognitively inactive lifestyle and age-related decline [10], and the extensive restrictions on contact imposed by COVID-19, especially with LTC facility residents, have increased the likelihood of this detrimental lifestyle condition [11]. Thus, there is a pressing need, particularly within LTC facilities, to establish an activating environment to prevent age-related decline.

This project explores a preventive training program targeting swallowing and linguistic-communicative skills, aimed at preserving the social participation of older individuals in LTC facilities in their everyday lives.

## Methods/design

A preventive intervention program, combining orofaciopharyngeal and linguistic-communicative components, will be implemented in LTC facilities in the greater Hanover area, aimed at prolonging residents’ joint consumption of meals and everyday communication. In more detail, the intervention objectives will be to: (a) activate sensitive and motor skills in the orofaciopharyngeal area to counter age-related swallowing disorders, and (b) stimulate communicative abilities, emphasizing semantic-lexical activation to counter age-related word retrieval disorders.

The cluster-randomized intervention study, utilizing a waiting group design, will aim at testing the following hypotheses:

### Main hypothesis

The orofaciopharyngeal and speech-communication program will help to prevent a decline of swallowing skills in older individuals.

### Secondary hypotheses

The orofaciopharyngeal and linguistic-communicative intervention will:

- improve language skills in older individuals;
- be positively accepted by older individuals within LTC facilities; and
- improve the social participation of older individuals, with respect to
  - everyday communication,
  - swallowing- and eating-related everyday activities, and
  - the preservation of cognitive abilities.

**Fig. 1:**
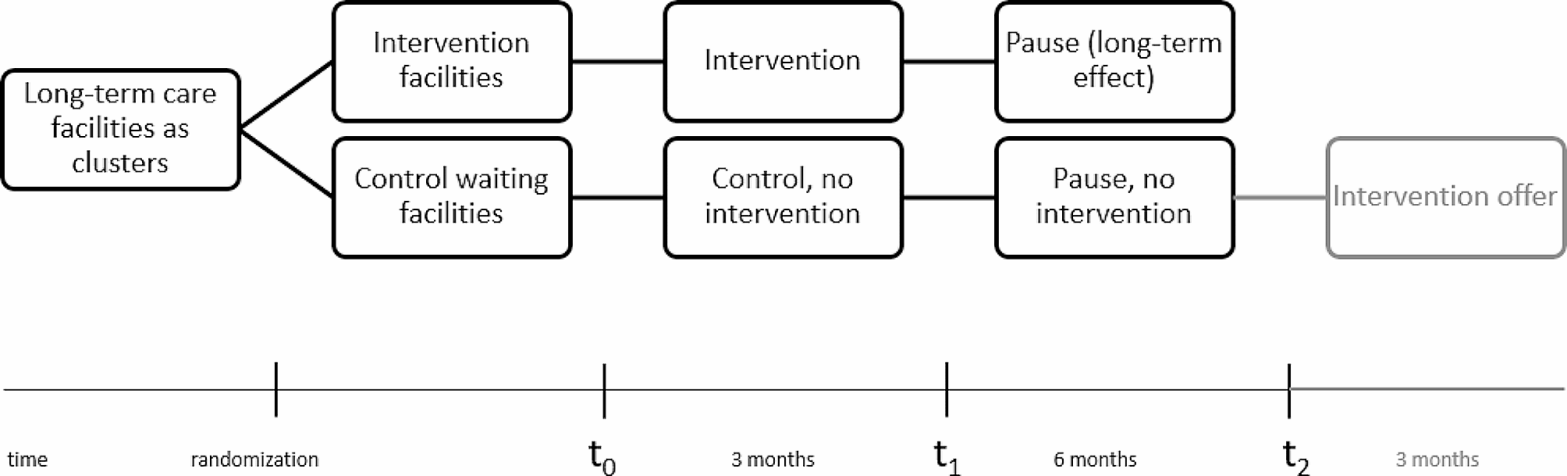
Schematic overview of the study design

The project design will follow an MRC phase III approach, based on the Medical Research Council’s (MRC) recommended staged model for complex interventions [12]. A cluster-randomized intervention study with a waiting group design will be conducted, measuring swallowing capacity using the Timed Test of Swallowing Capacity (TTSC) [13] as a primary endpoint. If the intervention proves successful, participants in the control waiting groups will be offered the opportunity to participate after t_2_ (Fig. 1).

### Characterization of LTC facilities as clusters and participants

The intervention will be implemented in 12 LTC facilities in a group setting, with approximately eight subjects per group. Recruitment will follow a two-stage process. In the first stage, LTC facilities will be recruited. For this purpose, the central supporting organization will contact relevant LTC facilities and members of the research team will introduce the project (i.e., contents, scope of participation, data protection, etc.). Subsequently, interested LTC facilities will be sent detailed project descriptions, to ensure their free, open, and informed participation. The final set of LTC facilities will be determined on the basis of their informed consent of unit managers, number of beds, sponsorship structure (in Germany, facilities are run by profit (i.e., private) or non-profit (e.g., municipal or religious) organizations), and geographical location.

The following inclusion criteria will apply to LTC facilities: (i) located in the greater region of Hanover, (ii) structured by diverse sponsorships, and (iii) providing a regular range of activating group activities (e.g., movement and cognitive training as part of “usual care”). Gerontopsychiatric LTC facilities, facilities for the disabled, and exclusive day care facilities will be excluded. In accordance with cluster randomization recommendations [13], the 12 selected LTC facilities will be stratified by size, sponsorship structure, and resident characteristics, and then randomly distributed to either the intervention or the control waiting group.

In the second step, LTC facility coordinators will recruit 6–10 resident participants from their facility. Prior to this selection, members of the research team will introduce the project within each facility. Participation will be voluntary and will only begin after participants have received detailed information about the study objectives, procedures, and possible consequences, as well as their participant rights. Participants will be able to withdraw their consent at any time. The inclusion criteria for participation will be as follows:

- aged 65 years or older and residing in an LTC facility;
- no mild or moderate frailty (i.e., Clinical Frailty Scale: level < 5), according to Rockwood [14] (as assessed by caring professionals);
- able to give informed consent (as assessed by caregivers and, if applicable, legal guardians); and
- sufficiently able to hear (i.e., 80% speech comprehension at an everyday speech volume).

The exclusion criteria will be as follows:

- a current diagnosis of a swallowing, speech, and/or language disorder and an associated prescription for speech, swallowing, or language therapy;
- suspected aspiration (i.e., EAT-10 score > 2);
- moderate or severe dementia (i.e., DemTect ≤ 8); and
- estimated life expectancy of < 6 months, based on the “Double Surprise Question” (as assessed by caring professionals) [15].

### Ethics

The OrkA project, developed in accordance with the Declaration of Helsinki, was approved by the Ethics Committee of Hannover Medical School, Germany (no. 10650_BO_S_2022) and registered with DRKS (German register of clinical trials) in June 2023 (study ID: DRKS00031594). Any modifications are required to be duly notified and approved in writing.

### Data collection

#### Sample size calculation

The primary outcome variable will be swallowing capacity, as measured via the Timed Test of Swallowing Capacity (TTSC). Based on a two-tailed unpaired t-test with a significance level set to 5% and a power of 80%, *n* = 5 clusters per group will be required (assuming *n* = 8 individuals per cluster). Anticipating a difference in the change in swallowing capacity of 2.8 ml/s between groups, with a pooled standard deviation of 3 ml/s [16] and an ICC of 0.1 [14, 15],^1^*n* = 10 clusters with a combined total of *n* = 80 individuals will be required.

Studies investigating individuals with pre-existing dysphagia in nursing homes [16] and nursing home residents, more generally [17], have reported dropout rates between 10 and 52%. According to the defined inclusion and exclusion criteria, the present study will address less vulnerable residents with no pre-existing dysphagia. Thus, a lower dropout rate of approximately 20% is assumed. On the basis of these assumptions, a sample size of approximately *n* = 100 participants in 10–12 clusters (i.e., 5–6 intervention facilities and 5–6 control waiting facilities) will be required.

#### The OrkA intervention

The preventive intervention will aim at maintaining and improving orofaciopharyngeal and linguistic-communicative competency in older individuals. It will span 12 weeks, with twice weekly 1-hour group sessions. Sessions will be conducted by trained speech and language therapists with experience caring for older patients and expertise in dysphagia and neurology. Each session will comprise two orofaciopharyngeal exercises and two semantic-lexical exercises within a communicative-stimulating environment. Participants may elect to complete voluntary homework assignments based on the contents of each session.

Orofaciopharyngeal exercises will encompass:

- orofacial perception (i.e., experiencing the oral cavity and oral stereognosis exercises);
- tonus and coordination training of oral cavity muscles;
- isotonic and isometric exercises of facial, lip, and tongue muscles;
- alternating movements;
- exercises for pharyngeal and veloparyngeal activation (including airflow control exercises, negative pressure exercises, and laryngeal elevation); and
- compensatory aspects, including upright posture, bolus preparation, and postural changes in swallowing techniques.

Semantic-lexical activation exercises will focus on topics relevant to participants’ everyday lives (relating to, e.g., family, seasons, and food), including:

- semantic feature analysis (e.g., categorization, sorting tasks, and identification of commonalities);
- lexical retrieval (e.g., analysis and synthesis tasks concerning word forms);
- part-whole relations, involving sequences of daily activities; and
- hyperonyms and hyponyms.

Given age-related decreases in naming accuracy and increases in word retrieval access time [4], these skills will be given special attention through incentivized quick naming (e.g., time competitions, group challenges). Subsequently, appropriately practiced word fields will be integrated into targeted biography-related communication [3, 18, 19].

Based on scientific findings, shared decision making will serve as a guiding principle [20]. Participants will be invited to choose the tasks they practice between sessions and post-intervention [21], with the aim of increasing intervention effectiveness and individual self-efficacy [21]. Studies have shown that rigid exercise frameworks tend to be less accepted and less effective than self-selected aims and content that the individual considers meaningful. Homework completion and success will be documented and discussed at the start of each session. In addition, participants will maintain training diaries, documenting their progress, questions, and problems.

Considering the vulnerability of the participant population, the intervention will be interrupted in the event of acute illness or a crisis-like exacerbation of chronic illness (e.g., apoplexy, aspiration pneumonia). In the event of a deterioration in food intake by more than one point according to the EAT-10, the intervention will be discontinued. An intermediate evaluation of swallowing ability (by means of the EAT-10) will be conducted to continuously ensure patient safety.

### Evaluation

Various evaluation tools will be utilized at three time points (t_0_, t_1_, t_2_) to test the working hypotheses. Assessments of frailty [14, 22] and hearing ability will only be collected at t_0_, to verify the inclusion and exclusion criteria. Table 1 shows the timing of all measurements of individual diagnostic criteria.

**Table 1:** Study overview regarding diagnostic measures and time points

time point	study period						
	enrollment	allocation	post-allocation			close-out	
	inclusion of LTC-facilities	statification randomization	pre-inter-vention (t_0_)	intervention	post-inter-vention (t_1_)	6-month follow up (t_2_)	intervention waiting group
**Enrollment**							
review of inclusion criteria of the LTC-facilities	X						
allocation of the LTC-facilities		X					
informed consent of the LTC-residents			X				
**Assessment**							
demografic and clinical data of the LTC-residents			X				
inclusion criteria of the LTC-residents: Clinical Frailty Scale			X				
inclusion criteria of the LTC-residents: speech audiometry			X				
*clinical assessments*							
cognitive ability: Dementia-Detection-Test (DemTect)			X		X	X	
language ability: Regensburg Word Fluency Test (RWT), semantic and phonological verbal fluency			X		X	X	
swallowing ability: Timed Test of Swallowing Capacity (TTSC)			X		X	X	
Questionnaire about every day Communication Practice– Communicative Activity Log (CAL)			X		X	X	
quality of everyday swallowing: Eating Assessment Tool (EAT-10)			X	X	X	X	X
*process evaluation*							
notes and protocols of the intervention process diary of the Residents				X			
evaluation of the intervention: feedback questionnaire for residents					X		
**intervention group**				X			
**control-waiting group**							X

#### Description of the study process

The OrkA project will be divided into three main phases: In phase 1, the intervention program will be prepared and the 12 LTC facilities in the project region will be recruited. Phase 2 will involve the recruitment of test subjects, administration of the status survey t_0_ in participating LTC facilities, implementation of the intervention in the participating facilities, and the follow-up survey t_1_. Subsequently, the follow-up survey t_2_ will be administered 6 months after the intervention, coinciding with the implementation of the intervention in the three control waiting facilities. In this second project phase, processes (i.e., the intervention with groups A and B, the intervention and testing with control waiting groups) will be staggered, due to the extensive scope of personnel work and process-related adjustments. This will ensure that the intervention can be carried out with the planned personnel (i.e., two speech language therapists), thereby minimizing any influence of personnel factors on the final results. Moreover, the process evaluation will allow for initial adjustments to be made, in the event that individual components (e.g., the control waiting design) lead to– for example– a high dropout rate.

Phase 3 will involve the summary process and evaluation of the intervention outcome. Table 1 shows the steps and time schedule.

#### Data processing and statistics

Data collected in the LTC facilities, test results, and data from the structured surveys will be saved (i.e., input) and analyzed using the Statistical Package for the Social Sciences for Windows (SPSS Inc., Chicago, IL/USA), in the version currently available at the Institute. The data will be stored on the secure servers of Hannover Medical School, and access to the data will be limited to the research team. For quality assurance, random checks of every fifth entry in the test protocols and plausibility checks will be carried out. Given the low risk of the preventive intervention, no external data monitoring committee is deemed necessary.

As part of the process analysis, meeting minutes and free-text answers to the surveys will be transcribed and pseudonymized. The research team are familiar with the recommendations for good scientific practice in qualitative research, with respect to data protection [23]. Any missing values will be replaced sensibly and conservatively.

Statistical significance will be determined by *p*-values < 0.05. Descriptive statistics will be employed to define frailty, hearing, swallowing, speech, cognitive abilities, and changes over time. A descriptive analysis will be conducted regarding the participating LTC facilities, the intervention study population, and the control waiting group. Additionally, a comparative analysis of central characteristics (i.e., gender distribution, age, frailty, etc.) will be carried out.

The primary endpoint will be evaluated at the individual level, according to the intention-to-treat principle. To detect statistically significant differences in the change in TTSC between groups at t_0_, t_1_, and t_2_, an unpaired t-test will be conducted with a two-sided significance level of 5%. Given a *p*-value < 0.05, any difference will be deemed statistically significant.

For the exploratory analysis of the secondary endpoints of language ability, subjective assessment of swallowing ability (according to the EAT-10), and communicative practice (according to the CAL), a comparative analysis will be conducted between the intervention and control waiting groups at t_0_, t_1_, and t_2_, using t-tests for unconnected samples [309]. A separate comparative analysis of the endpoints between measurement time points within the intervention group and the control waiting group will be conducted, using t-tests for connected samples.

Univariate and bivariate statistical procedures, as well as multivariable regression analyses (i.e., linear and logistic), will be used to assess possible associations between different dependent and independent variables, such as the influence of the LTC facility or sex. Continuous variables will be presented as means and standard deviations, while categorical variables will be presented as median and interquartile ranges.

The process evaluation will be based on an analysis of the documented frequency or continuity of active participation in the training units, the frequency of self-training, and the survey, which will be primarily evaluated quantitatively using univariate statistical methods (e.g., frequency counts, measures of central tendency, dispersion). In addition, subgroup analyses using bivariate statistical methods are planned (e.g., examination of a connection between participation frequency or dropout rate and the presence of mild to moderate frailty or dementia). Field notes, short minutes of sessions, and free-text information in the survey will undergo content analysis and topic evaluation [24].

## Discussion

This study protocol describes the design of a multidisciplinary, single-center, cluster-randomized intervention study with a waiting group design, conducted within LTC facilities in Lower Saxony, Germany.

Important aims of the project include:

- investigating the feasibility and effects of a preventive training program on swallowing and communicative capabilities;
- preserving social participation in the daily lives of older LTC facility residents;
- maintaining meals as a social and interactive event for as long as possible;
- activating sensitive and motor skills in the orofaciopharyngeal area to counter age-related swallowing disorders; and
- stimulating communicative abilities, with a focus on semantic-lexical activation to maintain linguistic abilities.

### Strengths and limitations

Currently, no preventive group intervention program for swallowing and speech competence is available to older individuals in LTC facilities in Germany. Individual therapy is prescribed only in the event of a diagnosed disorder (e.g., dysphagia, aphasia), typically at significant cost to the individual. To our knowledge, no prior study has investigated the effectiveness of preventive intervention programs for swallowing and speech competence among older individuals. However, previous studies investigating group intervention programs for LTC facility residents (e.g., physiotherapeutic muscle training, cognition training) have shown positive effects for muscular deterioration (e.g., sarcopenia) [22, 25] and cognition and language [6]. Likewise, group intervention programs for the treatment of dysphagia and aphasia in LTC facilities have been positively evaluated [26].

The results of this project are expected to provide important insights into the effectiveness of preventive programs to maintain speech, language, and swallowing abilities in older individuals, which might support the establishment of care guidelines. Positive long-term effects are also expected through the participatory design and the teaching of safe swallowing and word retrieval strategies, which will enable residents to continue improving their swallowing and speech skills post-intervention.

It is possible that some limitations of the research will affect the results. In particular, recruitment poses a potential challenge, given reported high dropout rates of up to 52% in studies of LTC facility residents and dysphagic patients, more generally [27]. However, the defined inclusion and exclusion criteria aim at excluding more vulnerable individuals (i.e., with pre-existing dysphagia), thereby mitigating potential dropout due to illness or death. Nevertheless, it is possible that a high dropout will be observed due to high frailty. A significant proportion of LTC facility residents may be too frail to benefit from or participate in the proposed intervention program, as current data show that individuals tend to stay in their own home for as long as possible before moving into an LTC facility [8]. Once this move has been made, residents often require therapy, rather than preventive measures. Thus, it is possible that the target population for the proposed intervention will need to be recruited elsewhere (i.e., from senior centers, rather than LTC facilities).

Quite possibly, facilities that are more generally committed to providing extraordinary care and offering “more” to their residents will be more likely to participate in the study. Furthermore, the intervention program will be geographically limited to the Hanover area, raising questions about the generalizability of the results to LTC facilities located in other regions or federal states.

### Assessment tools and potential sources of measurement bias

Both clinical and self-reported assessment tools will be employed to capture possible changes on all levels. The Timed Test of Swallowing Capacity (TTSC) is a validated screening tool that is used to identify dysphagia and determine swallowing volume and speed. This tool was chosen for the present study, as the participants will be healthy subjects presenting with, at most, presbyphagia. As the TTSC measures specific functions, rather than focusing on symptoms of dysphagia, it is sensitive enough to also detect changes in the swallowing of healthy subjects.

The Dementia-Detection-Test (DemTect) is a normed psychometric tool that measures different cognitive functions to detect early-stage dementia. Previous research has verified its high interrater reliability, sensitivity (97%), and specificity (93%), establishing it as a fast and economic, yet sensitive tool for identifying even mild dementia.

The Regensburg Word Fluency Test (RWT) is a standardized and normed neuropsychological tool that assesses executive functions of the active vocabulary. High interrater and test-retest reliability have been reported. Two subtests measure phonological and sematic word retrieval, respectively.

As relationships will develop between the individual leading the intervention program and participants, measurement bias may arise during repeated measurements. Therefore, all evaluation measurements (t_0,_ t_1_, t_2_) and the delivery of the intervention program will be conducted by different members of the research team.

Several self-assessment tools will be used, including the Eating Assessment Tool 10 (EAT-10) and a slightly modified version of the Questionnaire about Everyday Communication Practice– Communicative Activity Log (CAL). The EAT-10 [28] represents a validated and internationally used questionnaire capturing subjective swallowing dysfunctions. Its 10 questions explore respondents’ perceptions of swallowing and swallowing functions in different contexts.

In the event that any of the study measures carries a risk of aspiration difficulty, severe hearing problems, or dementia in participants, consultation with the medical departments of the research team will be offered.

Of note, increased knowledge and awareness of the act of swallowing and speech production might heighten participants’ sensitivity, potentially leading to more critical self-assessment results at t_1_, compared to t_0_.

In addition to the above-described self-assessment tools, some additional questionnaires will be filled in by nursing staff: the Clinical Frailty Scale (CFS) [1, 4], to record patients’ degree of frailty (considering physical, cognitive, and psychological dimensions); and the “Double Surprise Question” [29], to capture life expectancy.

Positive intervention results for swallowing functions and/or communication skills are expected to prompt the implementation and offer of preventive group training programs with respect to swallowing, speech, and language, not only in LTC facilities but also with older individuals, in general.

### Electronic supplementary material

Below is the link to the electronic supplementary material.

Supplementary Material 1

## Data Availability

The study data and forms (in the German language) are available from the corresponding authors upon reasonable request. Data from the participating LTC facilities are not publicly available due to data privacy protection regulations.

## Abbreviations

CAL: Communicative Activity Log
CFS: Clinical Frailty Scale
DemTect: Dementia-Detection-Test
DRKS: German register for clinical trials
EAT-10: Eating Assessment Tool
e.g.: exempli gratia
etc: et cetera
ICC: interclass correlation coefficient
i.e.: id est
LTC: long-term care
ml: milliliter
MRC: Medical Research Council
*n*: sample size
OrkA: orofacial and communicative activation in old age
*p*: probabilitas
RWT: Regensburg Word Fluency Test
s: second
SPSS: Statistical Package for the Social Sciences
t: test time
TTSC: Timed Test of Swallowing Capacity

## Footnotes

1 Statistical support given by the Department of Biometrics at Hannover Medical School.
